# Supplementary material for: Conditional chemoconnectomics (cCCTomics) as a strategy for efficient and conditional targeting of chemical transmission
Source: eLife. 2024 Apr 30;12:RP91927. doi: 10.7554/eLife.91927 (PMC11060718; doi:10.7554/eLife.91927)
Supplement: Figure 3—source data 3. [file elife-91927-fig3-data3.zip › Figure 3 source data3 part 1.zip!/Figure 3-Source data 3-part1/1063-13-CRISPResso_Report_8cz_fsdr.zip!/CRISPResso_on_1063_15/9.Alleles_frequency_table_around_sgRNA_cgtcgagcacatgacatcaa.pdf]

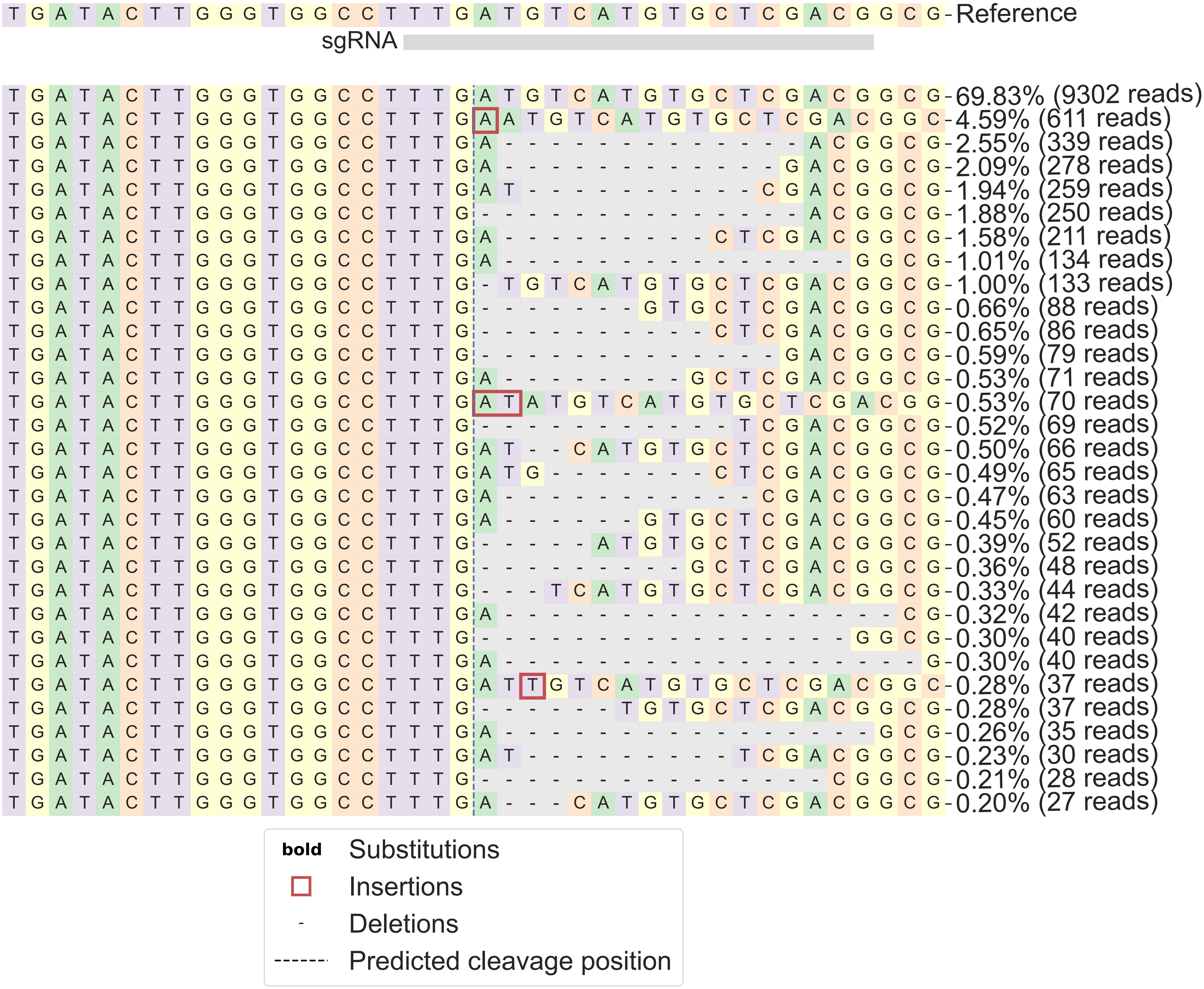
TGATACTTGGGTGGCCTTTGATGTCATGTGCTCGACGGCG Reference
sgRNA
TGATACTTGGGTGGCCTTTGATGTCATGTGCTCGACGGCG 69.83% (9302 reads)
TGATACTTGGGTGGCCTTTGAATGTCATGTGCTCGACGGC 4.59% (611 reads)
TGATACTTGGGTGGCCTTTGA-------------ACGGCG 2.55% (339 reads)
TGATACTTGGGTGGCCTTTGA------------GACGGCG 2.09% (278 reads)
TGATACTTGGGTGGCCTTTGAT----------CGACGGCG 1.94% (259 reads)
TGATACTTGGGTGGCCTTTG--------------ACGGCG 1.88% (250 reads)
TGATACTTGGGTGGCCTTTGA---------CTCGACGGCG 1.58% (211 reads)
TGATACTTGGGTGGCCTTTGA---------------GGCG 1.01% (134 reads)
TGATACTTGGGTGGCCTTTG-TGTCATGTGCTCGACGGCG 1.00% (133 reads)
TGATACTTGGGTGGCCTTTG-------GTGCTCGACGGCG 0.66% (88 reads)
TGATACTTGGGTGGCCTTTG----------CTCGACGGCG 0.65% (86 reads)
TGATACTTGGGTGGCCTTTG-------------GACGGCG 0.59% (79 reads)
TGATACTTGGGTGGCCTTTGA--------GCTCGACGGCG 0.53% (71 reads)
TGATACTTGGGTGGCCTTTGATATGTCATGTGCTCGACGG 0.53% (70 reads)
TGATACTTGGGTGGCCTTTG-----------TCGACGGCG 0.52% (69 reads)
TGATACTTGGGTGGCCTTTGAT--CATGTGCTCGACGGCG 0.50% (66 reads)
TGATACTTGGGTGGCCTTTGATG-------CTCGACGGCG 0.49% (65 reads)
TGATACTTGGGTGGCCTTTGA-----------CGACGGCG 0.47% (63 reads)
TGATACTTGGGTGGCCTTTGA------GTGCTCGACGGCG 0.45% (60 reads)
TGATACTTGGGTGGCCTTTG-----ATGTGCTCGACGGCG 0.39% (52 reads)
TGATACTTGGGTGGCCTTTG---------GCTCGACGGCG 0.36% (48 reads)
TGATACTTGGGTGGCCTTTG---TCATGTGCTCGACGGCG 0.33% (44 reads)
TGATACTTGGGTGGCCTTTGA-----------------CG 0.32% (42 reads)
TGATACTTGGGTGGCCTTTG----------------GGCG 0.30% (40 reads)
TGATACTTGGGTGGCCTTTGA------------------G 0.30% (40 reads)
TGATACTTGGGTGGCCTTTGATTGTCATGTGCTCGACGGC 0.28% (37 reads)
TGATACTTGGGTGGCCTTTG------TGTGCTCGACGGCG 0.28% (37 reads)
TGATACTTGGGTGGCCTTTGA----------------GCG 0.26% (35 reads)
TGATACTTGGGTGGCCTTTGAT---------TCGACGGCG 0.23% (30 reads)
TGATACTTGGGTGGCCTTTG---------------CGGCG 0.21% (28 reads)
TGATACTTGGGTGGCCTTTGA---CATGTGCTCGACGGCG 0.20% (27 reads)
bold Substitutions
Insertions
Deletions
Predicted cleavage position
